# Perioperative Management of Sickle Cell Disease in Complex Congenital Cardiac Surgery: A Compilation of Two Cases

**DOI:** 10.7759/cureus.30479

**Published:** 2022-10-19

**Authors:** Kunal A Soni, Vishal V Bhende, Tanishq S Sharma, Hardil P Majmudar, Amit Kumar, Bhadra Y Trivedi, Gurpreet Panesar, Kartik B Dhami, Manish Tiwari, Sohilkhan R Pathan

**Affiliations:** 1 Cardiac Anesthesiology, Bhanubhai and Madhuben Patel Cardiac Centre, Shree Krishna Hospital, Bhaikaka University, Karamsad, IND; 2 Pediatric Cardiac Surgery, Bhanubhai and Madhuben Patel Cardiac Centre, Shree Krishna Hospital, Bhaikaka University, Karamsad, IND; 3 Pediatric Cardiac Intensive Care, Bhanubhai and Madhuben Patel Cardiac Centre, Shree Krishna Hospital, Bhaikaka University, Karamsad, IND; 4 Pediatric Interventional Cardiology, Bhanubhai and Madhuben Patel Cardiac Centre, Shree Krishna Hospital, Bhaikaka University, Karamsad, IND; 5 Cardiac Surgery, Bhanubhai and Madhuben Patel Cardiac Centre, Shree Krishna Hospital, Bhaikaka University, Karamsad, IND; 6 Clinical Research Services, Bhanubhai and Madhuben Patel Cardiac Centre, Shree Krishna Hospital, Bhaikaka University, Karamsad, IND

**Keywords:** sickle cell anemia, intraventricular septal defect, cortriatriatum sinistrum, exchange transfusion, cardiopulmonary bypass

## Abstract

Preoperative exchange transfusion is frequently recommended in patients with homozygous sickle cell anemia (homozygous SS) who undergo cardiopulmonary bypass to reduce the concentration of circulated sickle hemoglobin. The information regarding the ideal level of sickle hemoglobin for sickle cell disease (SCD) patients who require surgery is still divergent in the literature. We present the successfully managed cases of two children aged 11 months and three years with homozygous SS who underwent cardiopulmonary bypass for double-outlet right ventricle and cor-triatriatum sinistrum, respectively. In both cases, we performed preoperative blood and exchange transfusion, as well as strict intraoperative invasive monitoring. We also maintained normothermia, avoided hypoxia and acidosis, and offered effective pain management.

## Introduction

β-globin mutation (autosomal recessive mutation) distorts normal hemoglobin and results in sickle cell disease (SCD). This distortion changes the shape of red blood cells (RBC) to sickle-shaped with a rigid membrane rich in sickle hemoglobin (HbS). Whenever deoxygenated, the HbS forms polymers, leading to cellular injury, microvascular occlusions, and inflammation in the vascular endothelium [1].

In hemoglobinopathies, there are two main types of autosomal recessive inherited disorders, namely, SCD and thalassemia. About 5% of the world’s population carries a mutated hemoglobin gene. However, SCD incidence is most common in Afro-Caribbeans in addition to other regions with less incidence, such as the Middle East [2].

A potential fatal sickling crisis may occur due to hypothermia, low pH, hypoxia, or low blood perfusion in SCD patients who require cardiac surgery [3]. Hence, these patients need modified routine perioperative management strategies to ensure successful outcomes. Given the rare cases of open-heart surgery in SCD patients, the literature is sparse in this area. Due to the advancement in diagnostics, surgical techniques, and anesthetic management skills, there has been a considerable increase in the number of SCD patients who require cardiac surgery. Nevertheless, there is a strong need to evaluate the available management or propose a new one for such patients.

Our present report evaluates open-heart surgery outcomes in two SCD patients at our institution, with particular emphasis on early peri and postoperative complications (within 30 days), in addition to deaths (up to two years).

## Case presentation

Written informed consent to allow the use of patients’ data was obtained from the parents or guardians before surgery.

Case one

An 11-month-old male patient with a history of recurrent respiratory tract infections and poor weight gain (6.2 kg) was brought to our Pediatric Cardiac Outpatient Department.

Two-dimensional (2D) echocardiography suggested congenital cardiac heart disease with the following clinical features: double-outlet right ventricle (DORV), ventricular septal defect (VSD), and severe pulmonary arterial hypertension (PAH). In addition, the child was diagnosed with SCD according to HbS (63%), HbA (1.6%), and HbF (35%). The patient’s preoperative hemoglobin (Hb) was 9.2 g/dL, while other baseline investigations were within normal limits. On the day before the surgery, we transfused the patient with 100 mL of freshly packed cell volume (PCV) to raise his Hb level to 11.3 g/dL.

Case two

A 3-year-old female patient weighing 9.5 kg was admitted due to breathing difficulties. On admission, the patient was kept on milrinone infusion for severe right ventricular (RV) dysfunction. Additionally, she experienced on-and-off febrile episodes, which were managed with antipyretic medications. Using 2D echocardiography, the child was diagnosed with cortriatriatum, atrial septal defect (ASD), severe tricuspid regurgitation, moderate RV dysfunction, and severe PAH.

The child was incidentally diagnosed with SCD. She underwent preoperative diagnostic cardiac catheterization with oximetry, which indicated high pulmonary vascular resistance. Following supplementation with oxygen and sildenafil, the pulmonary vascular resistance was significantly reduced, with step-up features of some reversibility.

As part of the SCD evaluation, the Hb electrophoresis showed the following results: HbS (49.8%), HbA2 (3.5%), HbF (46.7%), and sickling test positive.

The details of the preoperative Hb electrophoresis of both patients are presented in Table 1. The details of surgery in both cases are presented in Table 2.

**Table 1:** Preoperative hemoglobin electrophoresis of both patients with sickle cell disease. Hb: hemoglobin

Parameters	Case one	Case two
HbS	63.3%	49.8%
HbA_2_	1.6%	3.5%
HbF	35.1%	46.7%
Sickling test	Positive	Positive

**Table 2:** Summary of surgical procedures performed for both patients. DORV: double-outlet right ventricle; VSD: ventricular septal defect; PAH: pulmonary arterial hypertension; ASD: atrial septal defect

Description	Case one	Case two
Diagnosis	DORV, VSD, normally related great arteries, severe PAH	Cortriatriatum sinistrum, ASD, severe tricuspid valve regurgitation, moderate right ventricle dysfunction, severe PAH
Surgery performed	Intraventricular tunnel repair using glutaraldehyde-treated pericardial patch committing the left ventricle to the aorta	Cortriatriatum excision + autologous pericardial patch closure of ASD

Intraoperative management

**Figure 1:**
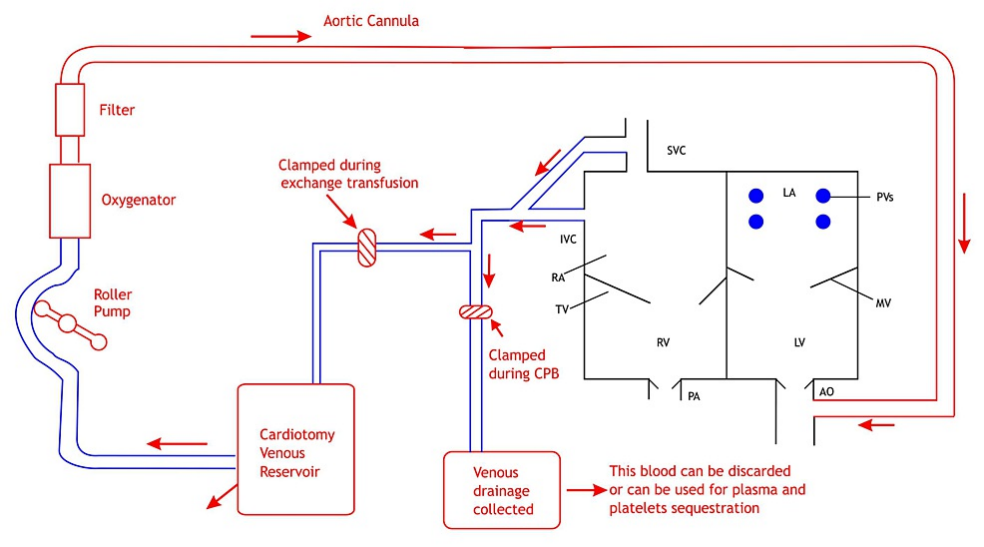
Cardiopulmonary bypass circuit showing arrangements for exchange transfusion. SVC: superior vena cava; IVC: inferior vena cava; RA: right atrium; LA: left atrium; PV: pulmonary vein; PA: pulmonary artery; AO: aorta; RV: right ventricle; LV: left ventricle; TV: tricuspid valve; MV: mitral valve

**Figure 2:**
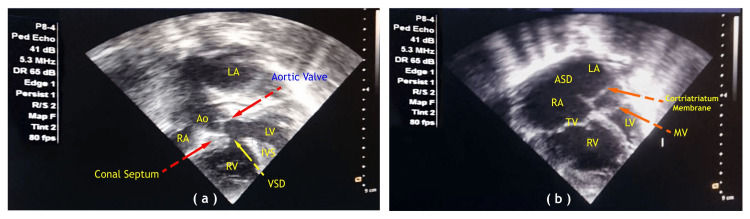
Preoperative 2D echocardiography apical four-chamber view showing (a) ventricular septal defect in case one and (b) cortriatriatum membrane in case two. RA: right atrium; LA: left atrium; ASD: atrial septal defect; AO: aorta; RV: right ventricle; LV: left ventricle; TV: tricuspid valve; MV: mitral valve; IVS: interventricular septum; VSD: ventricular septal defect

The patient’s vital parameters were monitored. Induction of anesthesia was done with injection ketamine, injection midazolam, injection fentanyl, and injection vecuronium. Anesthesia was maintained with oxygen, air, sevoflurane, and intermittent doses of injection fentanyl, injection midazolam, and injection vecuronium. After induction, we performed an exchange transfusion (Figures 1, 2).

Table 3 presents the strategy for the management of the two patients.

**Table 3:** Strategy of exchange transfusion.

Serial number	Description
01	Normothermia was maintained with warm fluids, warming blankets, and warm ambient temperature
02	After induction, a blood transfusion with 100 mL fresh packed cell volume was performed
03	Cardiopulmonary bypass management: after cannulation, a separate roller pump was used to perform a controlled exchange transfusion of 100 mL/kg over 10 minutes with simultaneous infusion of prime volume at the same rate to avoid hypotension
04	Sample was sent for hemoglobin lectrophoresis: HbS 9.1%, HbA 85.3%, HbF 3.1% (case one)
05	Normothermic cardiopulmonary bypass (temperature >34°C at all times) with warm blood cardioplegia. pH was maintained >7.4, and pO_2_ >300 mmHg
06	No vasoconstrictor was used on cardiopulmonary bypass
07	The surgery was uneventful in both cases. No episodes of hemolysis came off the cardiopulmonary bypass with milrinone and adrenaline inotropes
08	Modified ultrafiltration was performed in both cases
09	No blood transfusions were performed during the postoperative period in both cases
10	Pain relief by intravenous dexmedetomidine infusion and injection of paracetamol

Both patients had a smooth and uneventful postoperative clinical course. The 2D echocardiography in both cases revealed the correction of the cardiac lesions with good bi-ventricular function*. On discharge, Hb level (%) was 12.6 g/dL (case one) and 11.1 g/dL (case two). The two patients were followed up for two years (Table 4).

**Table 4:** Intraoperative and postoperative parameters of the two cases of sickle cell disease.

Parameters	Case one	Case two
Age	11 months	3 years
Sex	Male	Female
Weight	6.2 kg	9.5 kg
Cardiopulmonary bypass time	113 minutes	72 minutes
Aortic cross-clamp time	78 minutes	47 minutes
Flow rate L/minute	0.68–1.37 L/minute	0.86–1.73 L/minute
Mean blood pressure	60–70 mmHg	60–70 mmHg
Temperature	>34°C	>34°C
Activated clotting time	>480 seconds	>480 seconds
Ventilator hours	6 days	27 hours (one day)
Hospital stay	14 days	21 days
Late complications/Deaths (up to two years)	--	--

## Discussion

SCD is an autosomal recessive condition caused by a single-nucleotide polymorphism in the β-globin gene (point mutation) and characterized by hemoglobin SS. Under certain circumstances, SS molecules are polymerized, which distorts the erythrocyte membrane and leads to the characteristic sickle shape. Alteration in cellular transit through the microvasculature is mainly due to changes in the shape. Hypoxia, hypothermia, acidosis, or low blood flow lead to the accumulation of RBCs, resulting in vascular occlusion and thrombosis in many organs. These factors are common during cardiac surgery. Chronically, SCD can cause multiorgan injuries due to recurrent hemolytic anemia and vascular obstruction [4].

Yousafzai et al. previously reported a safe surgical procedure for heart valve and congenital heart diseases in SCD and sickle cell trait patients; however, the survival rates and surgical outcomes were promising [5]. Bocchieri et al., on the other hand, proposed a complete intraoperative exchange transfusion, in which HbS reduced to a level below 5% without any preoperative transfusions. In addition to hemoconcentration, plasma and platelet fractions were separated intraoperatively from the patient’s native RBCs to reduce the need for RBCs and clotting factor transfusion after the procedure [6].

To reduce the risk of sickling associated with cardiopulmonary bypass (CPB), Usman et al. proposed using a low-flow state, cold cardioplegia, and aortic cross-clamping, in addition to a minimally invasive and warm-beating heart approach [7]. Maddali et al. reported a case of an SCD patient who underwent an elective coronary artery bypass graft surgery using CPB techniques preceded by preoperative transfusions to increase the HbA levels above 60% [8]. In our SCD patients, we used preoperative blood transfusions to raise the hematocrit level while lowering the HbS levels [9,10]. Although there is no agreement on the safe HbS level before the surgery, some reports suggest an HbS level of less than 30% for major surgeries [11] or less than 5% for cardiac surgeries, either pre or intraoperatively [12].

Before the surgery, our patients had HbS levels above 30%. However, exchange transfusion can decrease HbS levels and increase HbA levels, cellular oxygen delivery, and hematocrit levels. Therefore, we used it to enhance perfusion and reduce the incidence of perioperative sickling crisis and multiple-organ injuries as cardiac surgery is associated with many predisposing factors.

In our cases, hypothermia was avoided by implementing normothermic CPB, warm air blankets, and preserving the temperature of the operation theater. To prevent hypoxia, we implemented a hyperoxygenating pump prime and administered fresh donor blood to maintain hematocrit >24% (sampled within 24 hours) and venous oxygen saturation >80%. However, pH was preserved in a normal range by monitoring arterial blood gas. In the present cases, we used warm cardioplegia and oxygenated crystalloid solution to deliver high PaO_2_ to the myocardium, improve the distribution of cardioplegia solution, and reduce local anoxia.

Continuous hemofiltration and modified ultrafiltration were used to reduce the inflammatory response and improve lung compliance. We neither used cell saver nor auto-transfusion pre nor postoperatively. Cardiac surgery is stressful, especially in pediatric patients; thus, sufficient analgesia and sedation are essential. Therefore, the patient received dexmedetomidine infusion as a sedative drug and intravenous paracetamol as analgesia. We also considered the opioid sedative effect on the respiratory system during the operation [13].

## Conclusions

Successful management of congenital cardiac surgery in the case of SCD deserves special consideration. Preoperative stabilization of patients regarding optimization of baseline Hb; initial exchange transfusion to minimize HbS levels; avoidance of intraoperative hypothermia, acidosis, hypoxia, and vasoconstrictor agents; and adequate pain relief with judicious use of sedatives make it a safe procedure. A team approach involving the pediatric cardiac surgeon, cardiac anesthesiologist, perfusionist, and intensivist is vital for the management of such cases.
